# CD10-Equipped Melanoma Cells Acquire Highly Potent Tumorigenic Activity: A Plausible Explanation of Their Significance for a Poor Prognosis

**DOI:** 10.1371/journal.pone.0149285

**Published:** 2016-02-16

**Authors:** Junna Oba, Takeshi Nakahara, Akiko Hashimoto-Hachiya, Min Liu, Takeru Abe, Akihito Hagihara, Takehiko Yokomizo, Masutaka Furue

**Affiliations:** 1 Department of Dermatology, Graduate School of Medical Sciences, Kyushu University, Fukuoka, Japan; 2 Department of Biochemistry, Juntendo University School of Medicine, Tokyo, Japan; 3 Department of Health Services Management and Policy, Graduate School of Medical Sciences, Kyushu University, Fukuoka, Japan; University of Connecticut Health Center, UNITED STATES

## Abstract

CD10 has been widely used in cancer diagnosis. We previously demonstrated that its expression in melanoma increased with tumor progression and predicted poor patient survival. However, the mechanism by which CD10 promotes melanoma progression remains unclear. In order to elucidate the role of CD10 in melanoma, we established CD10-overexpressing A375 melanoma cells and performed DNA microarray and qRT–PCR analyses to identify changes in the gene expression profile. The microarray analysis revealed that up-regulated genes in CD10-A375 were mostly involved in cell proliferation, angiogenesis, and resistance to apoptosis; down-regulated genes mostly belonged to the categories associated with cell adhesion and migration. Accordingly, in functional experiments, CD10-A375 showed significantly greater cell proliferation *in vitro* and higher tumorigenicity *in vivo*; CD10 enzymatic inhibitors, thiorphan and phosphoramidon, significantly blocked the tumor growth of CD10-A375 in mice. In migration and invasion assays, CD10-A375 displayed lower migratory and invasive capacity than mock-A375. CD10 augmented melanoma cell resistance to apoptosis mediated by etoposide and gemcitabine. These findings indicate that CD10 may promote tumor progression by regulating the expression profiles of genes related to cell proliferation, angiogenesis, and resistance to apoptosis.

## Introduction

Melanoma is one of the most life-threatening malignancies of the skin and mucosa, and represents a complex and heterogeneous disease. To improve its overall clinical outcome, numerous studies have been performed to identify prognostic markers such as p16, p53, cyclin A, Ki-67, Bcl-2, MCAM, CXCR4, and metallothionein in melanoma [1, 2]. We also reported that tumor expression of CD10 [3], MFG-E8 [4], and PD-L1 [5] could predict tumor progression and poor patient survival in melanoma.

CD10, a common zinc-dependent metalloendoprotease that cleaves and inactivates a variety of signaling peptides, is now widely known for its involvement in various cancers [6, 7]. Its role in tumor progression, however, seems to differ in a tissue- or cancer-specific manner [7]. Previous studies showed that CD10 is up-regulated in melanoma compared with that in benign nevi, with both tumor and stromal expression being reported [8, 9]. In addition, we demonstrated that the expression of CD10 in primary tumors can serve as an independent predictor of poor prognosis [3]. Although we found poorer prognoses in patients with melanoma with CD10 expression in primary tumors, we had still not determined the mechanism by which CD10 is involved in melanoma progression. From previous studies, we hypothesized that CD10 promotes melanoma progression by changing the phenotypic character of the cell, which helps it to survive. In order to determine the role of CD10 in melanoma progression, we performed analyses of the function and gene expression profile of CD10-equipped A375 cells by comparing them with mock-A375 cells.

## Materials and Methods

### Reagents

The phycoerythrin (PE)-conjugated anti-CD10 monoclonal antibody (mAb) used for flow cytometric analysis (clone HI10a) was purchased from BD (Franklin Lakes, NJ, US). The CD10 enzymatic inhibitors (phosphoramidon and thiorphan), anticancer drugs (etoposide and gemcitabine), and mitomycin C were obtained from Sigma-Aldrich (St. Louis, Missouri, US). Cell Counting Reagent SF for cell proliferation assay and G418 for the selection of transfected cells were purchased from Nacalai Tesque (Kyoto, Japan).

### Cell culture

The A375 human melanoma cell line was purchased from the American Type Tissue Culture Collection (Manassas, VA, US). Cells were cultured in a humidified atmosphere containing 5% CO_2_ at 37°C, and maintained in Dulbecco’s Modified Eagle’s Medium (Sigma Chemicals, Balcatta, WA, US) supplemented with 1% nonessential amino acids (Invitrogen, Carlsbad, CA, US), 1% HEPES buffer (Invitrogen), 5% fetal bovine serum (FBS; HyClone, Logan, UT, US), 100 μg/ml streptomycin and 100 IU/ml penicillin (GIBCO, Carlsbad, CA, US), and sodium pyruvate (GIBCO).

### Plasmid construction and stable transfection

The open reading frame of human CD10 (hCD10) was amplified from commercially available cDNA clones (DNAFORM, Yokohama, Japan) by PCR using a sense primer (5'-CTAGCTAGCATGGGCAAGTCAGAAAGTCAGATGG-3') covering an NheI site and an antisense primer (5'-CGGGGTACCTCACCAAACCCGGCACTTCTTTTCT-3') at the stop codon covering a KpnI site. The eukaryotic expression vector pCXN2.1 (+) [10] was used to drive the expression of inserted CD10 cDNA. The PCR product was subcloned into the NheI/KpnI site of pCXN2.1 (+) and designated pCXN2.1 (+)-hCD10. Accurate amplification of the insert was verified by DNA sequencing. A375 melanoma cells in six-well plates were transfected with 2.5 μg of plasmid DNA using Lipofectamine LTX (Invitrogen) in accordance with the manufacturer's protocol, and were selected with 0.3 mg/ml G418 (Nacalai Tesque) for two weeks. Cells were stained with PE-conjugated anti-hCD10 mouse IgG1 antibody (BD); cells overexpressing hCD10 were collected using a FACS Aria II Cell Sorter (BD Biosciences, San Jose, CA, US) in accordance with the manufacturer's protocol. After several passages with G418 selection, the expression of hCD10 in these cells was again confirmed by flow cytometric analysis as described previously [11]. Briefly, the cells were washed with phosphate-buffered saline (PBS), supplemented with 3 ml of PBS/2 mM EDTA, and then collected into new tubes. The cells were incubated with 2 μg/ml PE-conjugated anti-hCD10 antibody for 30 min at room temperature. After washing with PBS twice, the cells were suspended in 2% FBS containing PBS and subjected to the flow cytometric analysis. Successful stable transfection was also confirmed by Western blot as described below.

### Western blot analysis

Western blotting was performed using primary mouse monoclonal anti-CD10 antibody (1: 1000 dilution, Novocastra, New Castle, UK) as previously described [11]. Briefly, total protein was extracted from cultured cells using complete Lysis-M, EDTA-free (Roche Diagnostics GmbH Roche Applied Science, Mannheim, Germany) and the protein concentration was measured with a BCA protein assay kit (Thermo Scientific, Rockford, IL, USA). Sample proteins (20 ug) were separated by electrophoresis in NuPage 4–12% Bis-Tris gradient gels (Invitrogen), and then transferred to a PVDF membrane (Invitrogen). After blocking with 5% skim milk in TBS, the membrane was incubated with anti-CD10 antibody, followed by horseradish peroxide (HRP)-conjugated anti-mouse IgG secondary Ab (1: 1500 dilution) for 30 min. Signals were detected using SuperSignal^™^ West Pico Chemiluminescent Substrate (Thermo Scientific) and ChemiDoc^™^ Touch imaging System (Bio-Rad Laboratories, Hercules, CA, US). The same membrane was used to detect GAPDH (1:1000 dilution, Cell Signaling Technology, Danvers, MA, US) as loading controls.

### DNA microarray and qRT-PCR

Total RNA was isolated using an RNeasy Mini Kit (QIAGEN, Valencia, CA), and was purified by ethanol precipitation and dissolved in RNase-free water. This preparation was electrophoresed on an Experion System (Bio-Rad Laboratories), and the quality was confirmed by measuring the ratio of band intensity between 28 S and 18 S ribosomal RNA. The total RNA (250 ng) was converted to its biotinylated-cRNA in accordance with the manufacturer’s instructions (Illumina TotalPrep RNA Amplification Kit; Ambion, Carlsbad, CA, US). Briefly, the reverse transcription of extracted mRNA to first-strand cDNA was performed for 2 hours at 42°C, using an oligo (dT) primer bearing a T7 promoter and reverse transcriptase (ArrayScript; Ambion). To prepare second-strand cDNA, DNA polymerase I and ribonuclease H were added to the above reaction mixture, and the solution was further incubated for 2 hours at 16°C. After cDNA purification using a cDNA filter cartridge, the eluted cDNA was used as the template for *in vitro* transcription. This reaction was performed at 37°C for 14 hours in the presence of T7 RNA polymerase and NTP mix conjugated with biotin, yielding multiple copies of biotinylated antisense RNA to each mRNA in the sample. A total of 1.5 μg of biotinylated-cRNA was overlaid onto individual array spots of the human microarray chip (Illumina HumanHT-12 v4). The chip was hybridized at 58°C for 19 hours, washed, labeled with fluorescent reagent, and scanned using an array reader (BeadArray Reader; Illumina, San Diego, CA, US). The data on gene expression were compiled using Bead Studio software (Illumina).

In the microarray analysis, average normalization was performed using Illumina software (Genome Studio v 1.8). If normalized expression values were below 0.1, then we replaced these values with 0.1. Probes with a detection *P*-value > 0.01 in all samples were removed. Statistical analyses were performed using R software (http://www.R-project.org). We calculated *P*-values and fold changes between CD10-A375 and mock-A375 groups. The significance of differences between the two groups was estimated using the package samr in R. The significantly differentially expressed genes were defined as those with *P* < 0.01 in a two-class unpaired Significance Analysis of Microarrays (SAM) t-test and fold change > 2 or < 0.5 between the two groups.

A heat map was created using Mev4.6 for the 1,247 probes of genes significantly differentially expressed between CD10-A375 and mock-A375. The distance between the samples in the heat map was calculated using the Pearson correlation coefficient. Gene expression values were normalized by a Z-scaling method using a gene filter library with R. Gene Ontology annotation was assigned to significant genes identified by SAM using LSKB software (World Fusion Inc., Tokyo, Japan). The array data set was deposited in the Gene Expression Omnibus (series GSE62464).

Fifteen representative genes identified by microarray were validated using qRT–PCR with commercially available primers, as shown in Table 1. Total RNA was reverse-transcribed with a first-strand cDNA synthesis kit for RT-PCR (PrimeScript RT Reagent Kit; Takara Bio Inc., Shiga, Japan), in accordance with the manufacturer’s instructions. For all samples, 50 ng of cDNA was used for qRT-PCR analyses. The reverse-transcribed cDNA was then subjected to qRT-PCR (SYBR Premix Ex Taq; Takara Bio Inc.) and thermal cycling (Mx3000P Real-time qPCR Systems; Stratagene, La Jolla, CA). The reaction conditions were denaturing at 95°C for 30 seconds, followed by 40 cycles of denaturing at 95°C for 5 seconds, and annealing and extending at 60°C for 20 seconds. The level of mRNA expression was estimated from the fluorescence intensity relative to β-actin (ACTB).

**Table 1:** Primer sequences used for real-time RT-PCR.

Symbol	Accession No.	Primer Set ID
CDCP1	NM_178181	HA199789
NCAM1	NM_181351	HA150280
S100A1	NM_006271	HA140006
CTSL1	NM_145918	HA181205
CAMP	NM_004345	HA140794
FGFBP1	NM_005130	HA160915
CAV1	NM_001753	HA096896
NRP1	NM_003873	HA207771
CTSK	NM_000396	HA181777
CTLA4	NM_001037631	HA186912
IL1A	NM_000575	HA189662
TLR4	NM_138557	HA207352
CD96	NM_005816	HA117764
TIMP3	NM_000362	HA150753
CD10	NM_007287	HA086153
ACTB	NM_001101	N.A.

### *In vitro* cell proliferation assay

Using the transfected A375 cells, cell proliferation was analyzed using a water-soluble tetrazolium 8 (WST-8)-based colorimetric proliferation assay kit (Cell Counting Reagent SF; Nacalai Tesque). The cells were seeded in triplicate at a density of 5,000 cells in 200 μl of culture medium supplemented with 5% FBS in 96-well plates, incubated for 24, 48, 72, or 96 hours, and cell viability was assessed in accordance with the manufacturer's protocol. Briefly, cells were washed gently with PBS three times and non-adherent or dead floating cells were removed. The cell count reagent was added to each well and the plates were incubated at 37°C for 3 hours to allow the conversion of the reagent to formazan by mitochondrial dehydrogenase. Formazan was quantified by measuring the absorbance at 450 nm using a microplate reader (FlexStation 3; Molecular Devices, Tokyo, Japan).

### *In vivo* experiments

This study was carried out in strict accordance with the Fundamental Guidelines for Proper Conduct of Animal Experiment and Related Activities in Academic Research Institutions under the jurisdiction of the Ministry of Education, Culture, Sports, Science and Technology, Japan. All animal procedures were performed under isoflurane anesthesia, and all efforts were made to minimize suffering. All experiments were approved by the institutional Animal Care and Experiment Committee (Permit Number: A27-095-0), and by the Gene Modification Safety Committee (Permit Number: 24–35) of Kyushu University. BALB/c nu-nu athymic mice aged six to eight weeks old were purchased from Charles River Laboratories (Wilmington, MA, US). On day 7, the mice were injected with CD10-A375 or mock-A375 cells (5 × 10^5^). Semi-confluent CD10-A375 or mock-A375 cells were trypsinized and resuspended in 100 μl of PBS and then inoculated subcutaneously into the backs of mice. In order to minimize suffering, mice were given anesthesia using isofurane at the time of tumor cell inoculation. Tumor growth was monitored every three to four days by measuring the tumors in two dimensions using a caliper. Tumor volume was calculated using the following formula: π/6 × (larger diameter) × (smaller diameter)^2^, and compared between the two groups. Furthermore, to assess the effect of the inhibition of CD10 enzymatic activity on tumorigenic assay, mice were administered intraperitoneally with phosphoramidon (20 μg per mouse) or thiorphan (20 μg per mouse) on the day of CD10-A375 tumor injection. The treatment protocol followed the guidelines of animal experimentation of Kyushu University. At 28 to 34 days after tumor injection, all mice were euthanized using isoflurane, and tumor tissues were excised, fixed in 10% formalin, and embedded in paraffin sections. When we observed ulceration on the skin of mice, we euthanized the mice immediately after we took notes about our observations. Each group consisted of six mice, and the same experiments were performed at least three times.

### Cell migration assay

Cell mobility was investigated using the IncuCyte^™^ Imaging System (Essen BioScience, Ann Arbor, MI, US). Cells at a density of 50,000 cells/100 μl/well were seeded in an Essen Imagelock 96-well plate and incubated until confluence in a standard CO_2_ incubator at 37°C for 24 hours. Uniform wounds were made in each well with the 96-well WoundMaker (Essen BioScience). The medium was removed, and the cells were washed twice with PBS to remove floating cells and debris. The 96-well plate was then inserted into the IncuCyte FLR platform (which includes an automated microscope), and the cells were incubated with 100 μl of medium in the presence or absence of mitomycin C (10 μg/ml) to prevent cell proliferation. Photographs of the same area of the wound were automatically taken within the CO_2_ incubator using IncuCyte zoom software (Essen BioScience) at intervals of 3 hours, and the data were analyzed using the IncuCyte software package (Essen BioScience). Cell migration is expressed as the percentage of the gap relative to the total area of the cell-free region immediately after the wound was made. For each well, three randomly selected images were acquired, all experiments were carried out in triplicate, and the results were averaged.

### Cell invasion assay

*In vitro* cell invasion assay was performed using a basement membrane-coated CytoSelect^™^ 24-well Cell Invasion Assay Kit (8 mm pores, Colorimetric Format, Cell Biolabs, San Diego, CA, US) according to the manufacturer’s instruction. Briefly, CD10- or mock-A375 cells were serum-starved for 24 h. Next, the cells suspended with serum-free media (3 X 10^5^ cells/well) were seeded in the upper chamber separated from a lower chamber filled with media containing 10% FBS and incubated for 72 h. After noninvasive cells were removed, the invasive cells were stained and quantified by measuring the absorbance at 570 nm using a microplate reader (iMark^™^ Microplate Absorbance Reader, Bio-Rad Laboratories). All assays were carried out 3 times in triplicates.

### Apoptosis assay

Transfected A375 melanoma cells were plated in six-well plates until 90% confluence and then treated with cytotoxic agents overnight. The cells were cultured with etoposide (from 1 to 100 μM) or gemcitabine (from 10 to 100 μM). Cell death was determined by Annexin V and PI staining in accordance with the manufacturer’s instructions (MEBCYTO^®^ Apoptosis Kit; Medical & Biological Laboratories Co., Ltd., Nagoya, Japan), and analyzed by flow cytometry.

### Statistical analysis

All experiments were reproduced at least three times with different passages of the cell lines. The data of CD10 RNA expression levels are presented as the mean, and statistical analyses were performed using Student’s *t* test *or* Welch’s *t* test. Comparisons of the time course of *in vitro* cell proliferation assay, scratch assay, and *in vivo* tumorigenic assay between the two groups (CD10-A375 vs. mock-A375) were performed by two-way ANOVA. A *P*-value of less than 0.05 was considered significant. Analyses were carried out with SPSS software (version 11.0) and with GraphPad Prism Version 5 (GraphPad Software). Error values for the *in vitro* experiments are provided as the standard deviation (SD) and for the *in vivo* experiments as the standard error (SE).

## Results

### Transfection of CD10 resulted in alteration in the expression of many genes

**Fig 1:**
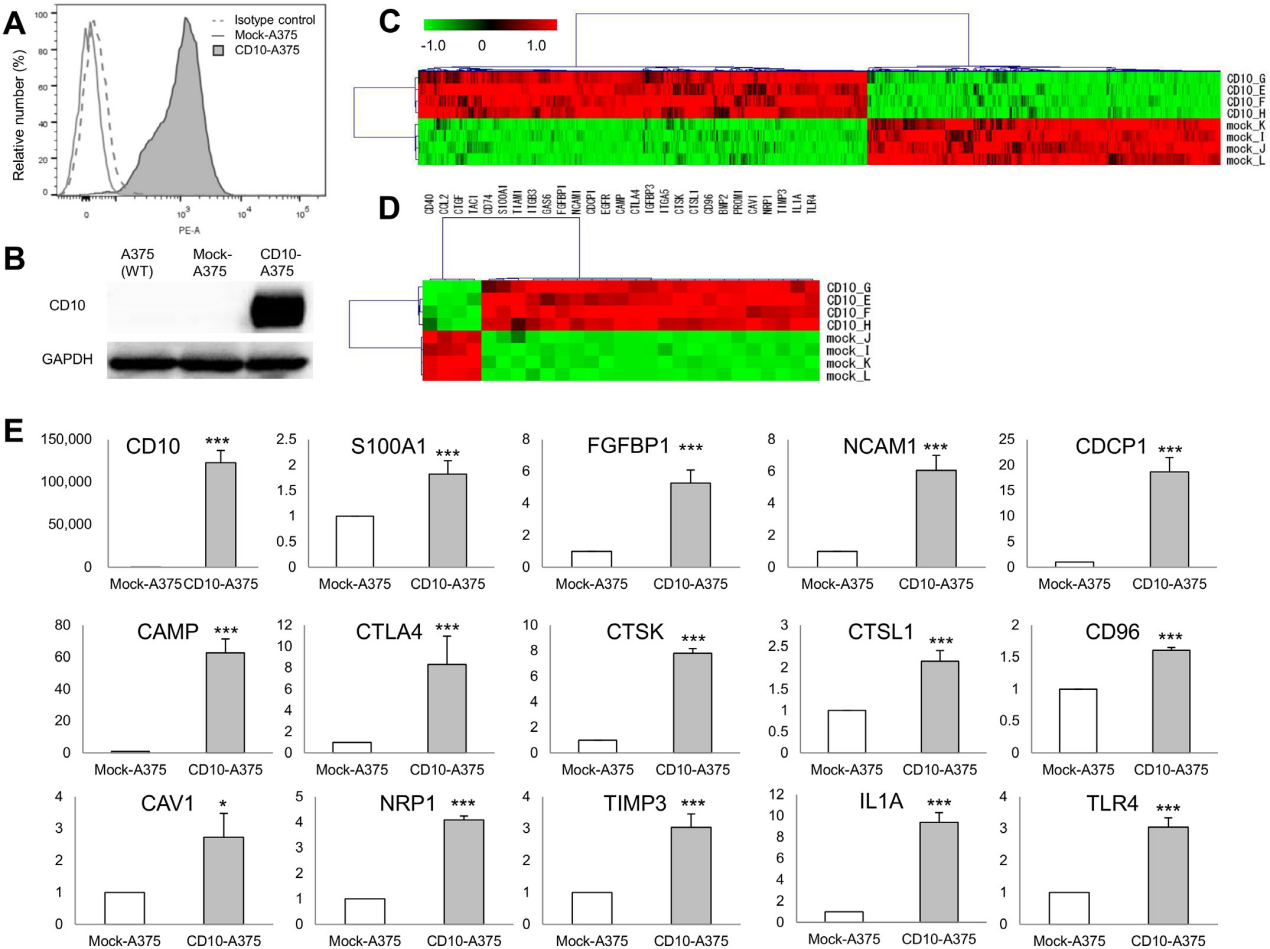
Establishment of CD10-A375 melanoma cells and gene expression profiles. (A) Stable transfection of CD10-A375 cells was confirmed by flow cytometry. After selection with G418, CD10-A375 or mock-A375 cells were examined for CD10 protein expression with PE-conjugated anti-hCD10 antibody. CD10-A375 exhibited marked surface expression of CD10 (gray shaded area), whereas mock-A375 did not (solid gray line). Isotype IgG control is shown with a gray dashed line. (B) Western blot analysis of CD10 and GAPDH in the wild type- and transfected- A375 cells. Successful transfection of CD10 was also confirmed in CD10-A375 cells by Western blot analysis. (C and D) Microarray analysis of gene expression in CD10-A375 vs. mock-A375 melanoma cells. (C) Heat map generated using MeV4.6 from the 1,247 probes of genes significantly differentially expressed between CD10-A375 and mock-A375 (*n* = 4 per group). A red color represents a higher-than-average standardized expression value, whereas a green color represents a lower-than-average one. Examples of differentially expressed genes are listed and sorted into major known functional categories in Table 2. (D) Heat map of the selected genes as shown in Table 2. (E) qRT–PCR validation analysis of representative genes that were significantly upregulated in CD10-A375 (*n* = 3). Each value was normalized for ACTB (β-actin), and the results are expressed as fold change over mock-A375. Primers used in the assay are shown in Table 2. *P < 0.05, **P < 0.01, ***P < 0.001; unpaired t test.

We first confirmed that the human melanoma cell line A375 does not express CD10 at the protein level. After A375 cells had been transfected with CD10, marked expression of the CD10 protein was observed in CD10-transfected A375 cells (CD10-A375), while empty vector-transfected A375 cells (mock-A375) displayed no CD10 expression (Fig 1A). Expression of CD10 protein in CD10-A375 was also confirmed by Western blot analysis as shown in Fig 1B. We next compared the gene expression profiles of CD10-A375 and mock-A375 by DNA microarray analysis. A total of 1,247 probes were shown to be differentially expressed between the CD10-A375 and mock-A375 cells, of which 698 were up-regulated and 549 were down-regulated in CD10-A375 by more than twofold, with a *P*-value of less than 0.01 (Fig 1C). A selected list of genes differentially expressed due to CD10 is shown in Table 2 and Fig 1D. Many genes regulating antiapoptosis, cell proliferation, angiogenesis, neoplasm metastasis, and inflammation were up-regulated in CD10-A375 compared with those in mock-A375. On the other hand, genes that are mainly associated with cell adhesion and cell migration were mostly down-regulated in CD10-A375 cells compared with those in mock-A375. To validate the accuracy of the microarray data, 15 representative genes with changes in expression of various levels upon CD10 transfection were chosen, and their expression levels were determined by qRT–PCR (Fig 1E). The qRT–PCR results showed an expression pattern similar to that of the DNA microarray, suggesting that the DNA microarray data obtained in the present study are valid.

**Table 2:** A list of differentially expressed genes in CD10-A375 cells compared with mock-A375 cells.

Main function	Gene symbol	Gene name	Accession no.	Ratio	P-value*
**Antiapoptosis**	*CDCP1*	CUB domain-containing protein 1	NM_022842.3	7.2	0.0016
			NM_178181.1	7.6	0.0011
	*ITGA5*	integrin, alpha 5 (fibronectin receptor, alpha polypeptide)	NM_002205.2	2.5	0.0005
	*NCAM1*	neural cell adhesion molecule 1	NM_000615.5	10.2	0.001
	*S100A1*	S100 calcium-binding protein A1	NM_006271.1	2.2	0.0024
	*GAS6*	growth arrest-specific 6	NM_000820.1	3	0.0022
**Cell proliferation**	*ITGB3*	integrin, beta 3 (platelet glycoprotein IIIa, antigen CD61)	NM_000212.2	2.2	0.0021
	*CD74*	CD74 molecule, major histocompatibility complex, class II invariant chain	NM_001025159.1	2.5	0.0029
	*CTSL1*	cathepsin L1	NM_145918.2	2.1	0.0005
	*PROM1*	prominin 1	NM_006017.1	31.9	0.0001
	*TIAM1*	T-cell lymphoma invasion and metastasis 1	NM_003253.2	2.6	0.0048
**Angiogenesis**	*BMP2*	bone morphogenetic protein 2	NM_001200.2	10.8	0.0001
	*EGFR*	epidermal growth factor receptor	NM_201282.1	3.1	0.0015
			NM_005228.3	5.7	0.0011
	*CAMP*	cathelicidin antimicrobial peptide	NM_004345.3	4316	0.0003
	*FGFBP1*	fibroblast growth factor-binding protein 1	NM_005130.3	34.8	0.0009
	*CAV1*	caveolin 1, caveola protein, 22 kDa	NM_001753.3	2.9	0.0005
	*NRP1*	neuropilin 1	NM_003873.4	73.9	0.0003
			NM_001024629.1	6.3	0.0007
**Inflammation/immune response**	*IGFBP3*	insulin-like growth factor-binding protein 3	NM_001013398.1	27.1	0.0005
			NM_000598.4	27.2	0.0002
	*CTSK*	cathepsin K	NM_000396.2	3.4	0.0003
	*CTLA4*	cytotoxic T-lymphocyte-associated protein 4	NM_005214.3	1225	0.0004
	*IL1A*	interleukin 1, alpha	NM_000575.3	113.8	0.001
	*TLR4*	toll-like receptor 4	NM_138554.2	10.3	0.0006
**Cell adhesion/migration**	*CCL2*	chemokine (C-C motif) ligand 2	NM_002982.3	0.1	0.0009
	*CD96*	CD96 molecule	NM_005816.4	6	0.0002
			NM_198196.2	5.6	0.001
	*CD40*	CD40 molecule, TNF receptor superfamily member 5	NM_001250.4	0.2	0.0039
	*TAC1*	tachykinin, precursor 1	NM_003182.1	0.4	0.0004
			NM_013996.1	0.3	0.0023
			NM_013997.1	0.1	0.0012
	*CTGF*	connective tissue growth factor	NM_001901.1	0.3	0.0001
			NM_001901.2	0.3	0.0013
	*TIMP3*	TIMP metallopeptidase inhibitor 3	NM_000362.4	2.4	0.0006

*p values were calculated using the package samr in R. The significantly differentially expressed genes were defined as those with P < 0.01 in a two-class unpaired Significance Analysis of Microarrays (SAM) t-test and fold change > 2 or < 0.5 between the two groups.

### CD10-overexpressing A375 cells showed higher cell proliferative capability *in vitro* and higher tumorigenicity *in vivo* than mock-A375

**Fig 2:**
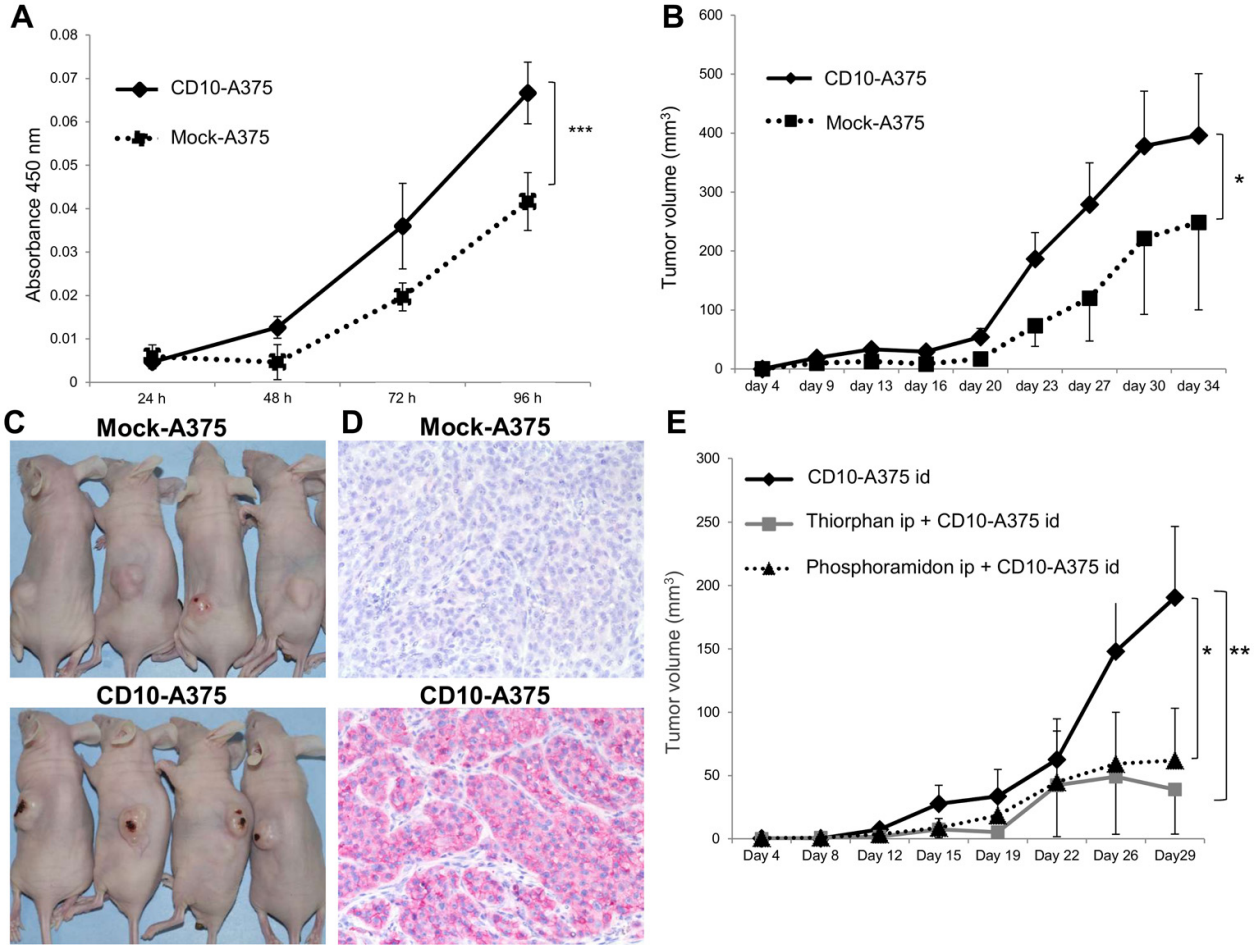
CD10 promotes *in vitro* cell proliferation and *in vivo* tumor growth, but its tumorigenic effect can be suppressed by CD10 enzymatic inhibitors. (A) CD10 expression promotes the proliferation of A375 melanoma cells *in vitro*. Growth curves of CD10-A375 (solid line) and mock-A375 (dotted line) cells grown in 5% FBS (*n* = 3 per group) show that CD10 expression increases cell growth. Cells were plated in triplicate in 96-well plates and incubated for 24, 48, 72, or 96 hours, and the number of viable cells was assessed, as described in Materials and Methods. Points, mean (*n* = 3); bars, SD. Cell proliferation rate was significantly higher in CD10-A375 (solid line) than in mock-A375 (dotted line). Representative data of three independent experiments are shown. (B) Effect of CD10 overexpression by A375 cells on tumor growth in a xenograft model. A total of 5 × 10^5^ CD10-A375 or mock-A375 cells resuspended in 100 μl of PBS were injected i.d. into the backs of BALB/C nude mice, and tumor growth was observed for about 28–35 days. Tumor diameters were measured and used to assess tumor volume, as described in Materials and Methods. Points, mean (*n* = 6); bars, SE. Each group consisted of six mice. CD10-A375 (solid line) manifested significantly higher tumorigenicity *in vivo* than mock-A375. Representative data of three independent experiments are shown. (C) Representative example of nude mice 30 days after tumor implantation, showing mice inoculated with CD10-A375 (right) or mock-A375 (left). It is noteworthy that mice injected with CD10-A375 tended to have ulceration on the tumor surface. (D) Immunohistochemical staining of a xenograft model for CD10. Upper, tumor from mock-A375-injected mouse; lower, tumor from CD10-A375-injected mouse. Staining is shown in red. Tumors from mice injected with CD10-A375 (lower) showed strong staining in tumor cells. Representative pictures of each group are shown. (E) Preventive effects of CD10 inhibitors on mouse tumor growth. Mice were injected i.d. with 5 × 10^5^ viable CD10-A375 cells resuspended in 100 μl of PBS with or without CD10 inhibitors. Phosphoramidon (20 μg/mouse) or thiorphan (20 μg/mouse) was administered intraperitoneally before the tumor cell injection. Points, mean (*n* = 6); bars, SE. Each group consisted of six mice. Both inhibitors significantly and markedly inhibited mouse tumor growth. Representative data of three independent experiments are shown. *P < 0.05; **P < 0.01; ***P < 0.001 by two-way ANOVA.

To examine the cell proliferative capability *in vitro*, the transfected A375 cells were harvested in triplicate in 96-well plates for 24, 48, 72, or 96 hours, and cell proliferation was assessed. The cell proliferation rate was significantly higher in CD10-A375 than in mock-A375 (*P* < 0.0001) (Fig 2A).

Next, we investigated whether CD10 had any effect on the formation and progression of tumors in xenograft models. It was noteworthy that mice injected with CD10-A375 developed significantly larger tumors than those injected with mock-A375 (Fig 2B). Additionally, mice injected with CD10-A375 tended to have tumors with ulcerations compared with those injected with mock-A375 (Fig 2C). The expression of the CD10 protein was confirmed by immunohistochemistry around 30 days after tumor inoculation (Fig 2D). We next examined the effect of CD10 inhibitors on mouse tumorigenicity using phosphoramidon and thiorphan. Mice were given phosphoramidon or thiorphan intraperitoneally, and then injected with CD10-A375; tumor growth was significantly suppressed in the CD10-inhibitor-treated group compared with that in the untreated group (*P* = 0.02 and *P* < 0.0001, respectively) (Fig 2E). We also examined the effect of CD10 inhibitors using mock-A375, but there was no significant difference in tumor growth between treated and untreated groups (data not shown).

### CD10-A375 showed lower cell mobility and cell invasion

**Fig 3:**
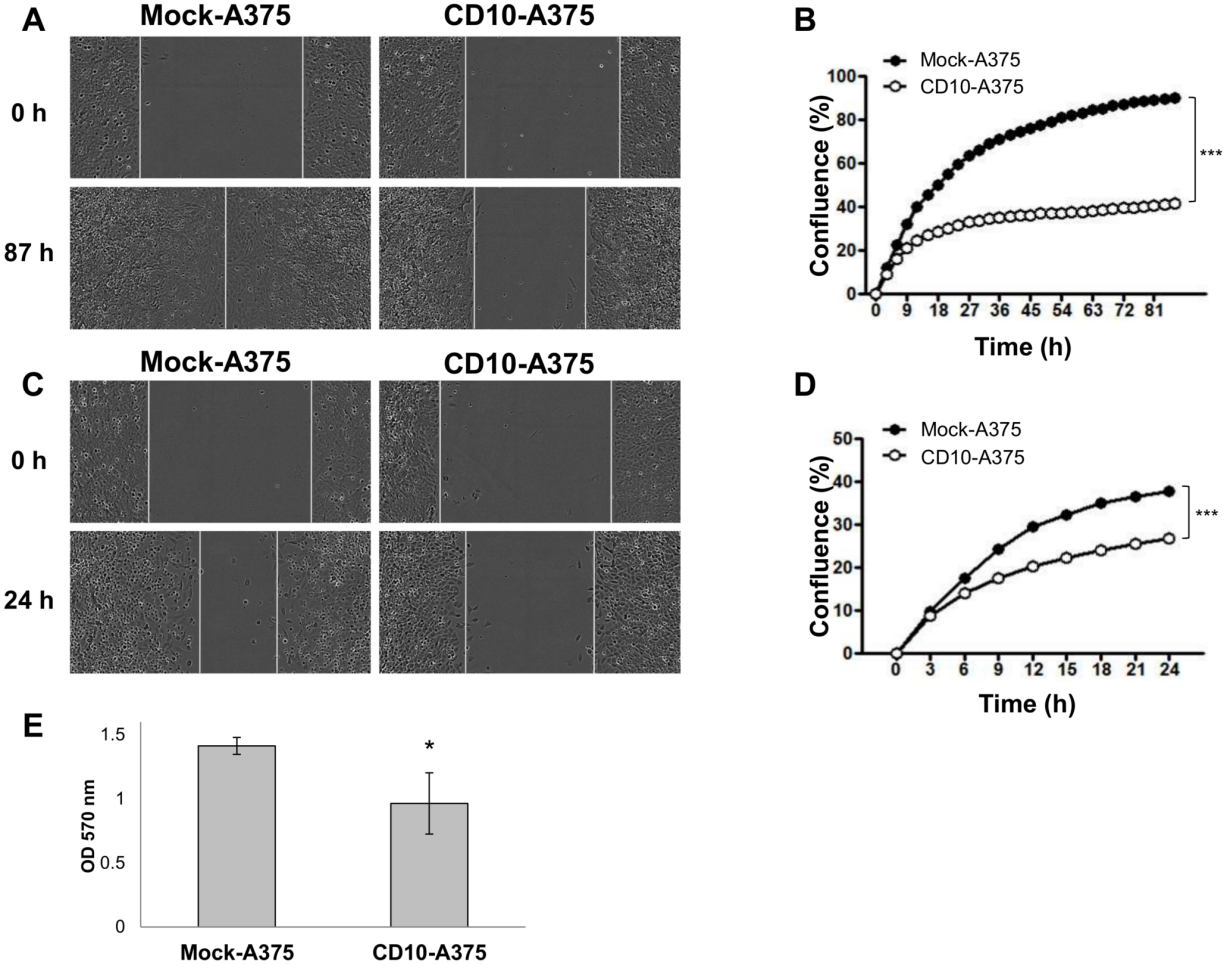
CD10 decreases cell migration and invasion. Cell migration assay (A-D) and invasion assay (E) of the CD10-A375 or mock-A375 cells were performed in the absence (A, B) or presence (C, D) of mitomycin C (10 μl/ml). (A and C) Representative images showing the wound gap filled by CD10-A375 (right) or mock-A375 cells (left) cultured in the absence of mitomycin C for 87 hours (A) or in its presence at a concentration of 10 μl/ml for 24 hours (C). (B and D) Graphs showing the time course changes in the percentage of wound healing after 87 hours of incubation without mitomycin C (B) or after 24 hours of incubation with mitomycin C (D). CD10-A375 (white dot) displayed lower migratory capacity both in the absence and in the presence of mitomycin C, compared with mock-A375 (black dot). ***P < 0.001 by two-way ANOVA. Results are representative of at least three independent experiments, and values represent averages of three independent measurements along the wound. (E) A graphic result of cell invasion assay of CD10-A375 vs. mock-A375 cells. Cells were plated on polycarbonate membrane inserts coated with uniform layer of basement membrane in 24-well plates (Cell Biolabs) and incubated for 72 hours. The invading cells were stained and quantified as indicated in the graph at 560 nm.

As cell mobility is one of the most important factors for cell invasion and migration, we had expected that CD10 overexpression would promote cell migration. However, as the DNA microarray results showed, CD10-A375 exhibited down-regulation of several genes that are mainly associated with cell adhesion and cell migration compared with mock-A375. To confirm this finding, we performed a cell migration assay to evaluate cell mobility using the IncuCyte^™^ Imaging System (Essen BioScience). CD10-A375 showed slower migration than mock-A375 (Fig 3A and 3B). In order to avoid the effect of cell proliferation, we administered mitomycin C, but the results were the same, as shown in Fig 3C and 3D.

Since we observed decreased migration in CD10-A375 compared with mock-A375, we next assessed the cell invasion using these cells. In accordance with the results of DNA microarray and migration assay, CD10-A375 exhibited lower invasion capability (Fig 3E, *P* = 0.0495). These results suggest that although CD10-A375 cells have higher cell proliferative and tumorigenic capability, they may have less migratory and invasion feature.

### CD10 overexpression resulted in higher resistance to anticancer drugs

**Fig 4:**
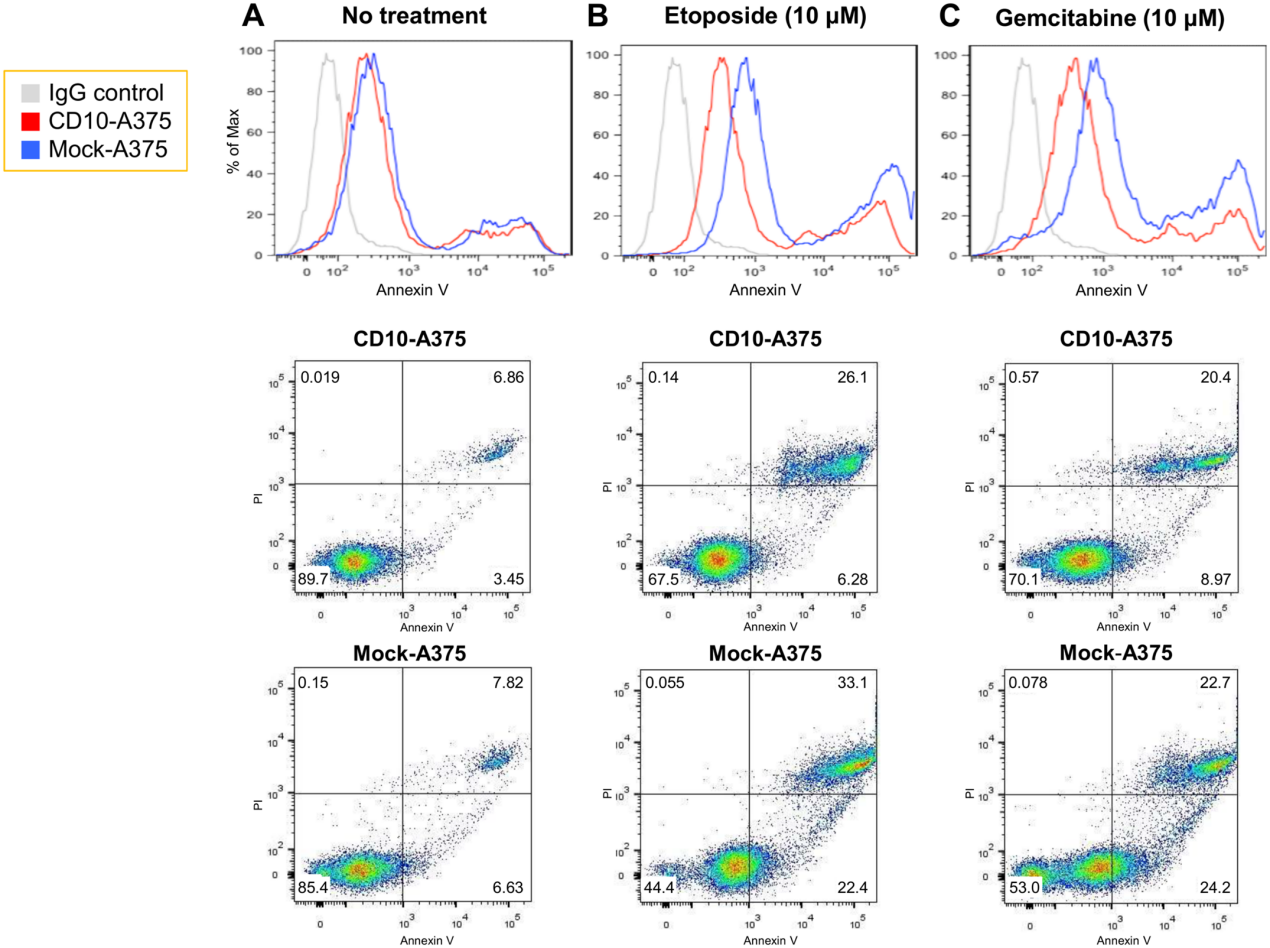
CD10 exhibits higher resistance to anticancer drugs. (A-C) Histogram (upper) and dot plot (lower) showing the proportion of Annexin V- and Annexin V/PI-positive cells 24 hours after treatment with anticancer drugs. CD10-A375 or mock-A375 cells were treated with etoposide (B) or gemcitabine (C) overnight. Cell were harvested and stained with Annexin V and PI, and 10,000 events were counted by flow cytometry. Without any treatment (A), the number of Annexin V-positive apoptotic or Annexin V/PI-positive dead cells was low and there was no difference between CD10-A375 and mock-A375. Under the treatment with the anticancer drugs, however, the number of apoptotic cells increased remarkably. CD10-A375 cells (red line) displayed a lower proportion of Annexin V-positive or Annexin V/PI-positive dead cells induced by anticancer drugs than mock-A375 (blue line), showing stronger resistance. Isotype control is shown with a gray line.

In clinical practice, not only the tumor cell characteristics (such as proliferative ability) but also resistance to therapeutics is critically associated with patient prognosis. Therefore, in order to determine the effect of CD10 on resistance to anticancer drugs, we performed an apoptosis assay using several cytotoxic anticancer drugs. When there was no treatment, the proportion of Annexin V-positive dead or apoptotic cells with or without PI positivity was limited and there was no difference between CD10-A375 and mock-A375 (Fig 4A). However, when we added anticancer drugs, such as gemcitabine and etoposide, the number of dead or apoptotic cells increased remarkably. Interestingly, CD10-A375 cells were more resistant to anticancer drugs than mock-A375, showing a much smaller number of apoptotic or dead cells (Fig 4B and 4C). These findings are in accordance with our DNA microarray analysis, where we observed many upregulated genes involved in antiapoptosis (Table 2).

## Discussion

For the past few decades, CD10 has been used as a diagnostic and prognostic marker (for both good and poor prognoses) in various cancers [7, 12]. Stromal CD10 expression was reported to correlate with poor prognosis in pancreatic cancer [13], breast cancer [14], and gastric carcinoma [15]. Similarly, tumor expression of CD10 has been reported to promote tumor progression in colorectal cancer [16], prostate cancer [17–20], bladder cancer [21], and melanoma [3]. On the other hand, CD10 has also been reported to inhibit tumor progression in cervical carcinoma [22] and ovarian cancer [23]. These previous reports indicate that CD10 has a variety of functions regarding tumor progression that exhibit a tissue-specific pattern. In this study, we aimed to elucidate how CD10 is involved in melanoma progression as a poor prognostic factor.

We observed a successful CD10 stable transfection in human melanoma cell line A375. DNA microarray analysis demonstrated that CD10-A375 cells displayed much more potent tumor-promoting features than mock-A375, with many up-regulated genes involved in antiapoptosis, angiogenesis, and cell proliferation. The present findings of qRT–PCR, cell proliferation assay, tumor xenograft tumorigenic assay, cell migration and invasion assay, and apoptotic assay were all consistent with the result of the DNA microarray analysis.

As for its localization in melanoma, CD10 protein expression can be observed mainly at the cell membrane but also in the cytoplasm of melanoma tumor cells as well as in stromal cells [3, 8, 9]. However, previous and our current studies suggest that CD10 in tumor cells are playing an important role in melanoma progression; in our previous study [3], we showed that tumoral expression of CD10 was significantly associated with worse prognostic factors such as higher Breslow thickness and ulceration, as well as shorter patient survival; our current *in vitro* cell proliferation assay and apoptosis assay as well as xenograft experimental study further support this idea by using CD10-transfected melanoma cells. Further, our flow cytometric analysis shows CD10 membranous expression in CD10-transfected A375 cells (Fig 1A). Although stromal expression of CD10 might also have biological effects in tumor microenvironment and there might be crosstalk or interaction between tumor and stromal CD10, our findings strongly indicate that CD10 in melanoma tumor cells itself has an essential role.

CD10-A375 showed greater cell proliferation than mock-A375 cells without adding any stimulation. There are several possible explanations for this: first, CD10-equipped A375 cells may have different biological features that make cell proliferation easier; and second, there might be a ligand or growth factor that binds CD10 and stimulates growth. Similarly, an *in vivo* experiment showed that mice injected with CD10-A375 tended to develop larger tumors with ulceration than those injected with mock-A375 (Fig 2B and 2C). Ulceration is one of the most important prognostic factors in melanoma [24], and the result that CD10-A375-implanted tumors tended to have ulceration compared with mock-A375-implanted tumors is interesting and reasonable. It is known that the enzymatic function of CD10 is inhibited by molecules containing biochemical domains such as thiol, carboxyl, hydroxamate, or phosphoramide with very high affinity for the zinc catalytic domain [25]. CD10 is also sensitive to phosphoramidon, a *Streptomyces* metabolite first identified as a thermolysin inhibitor that binds the active enzymatic site of CD10 [26]. It is noteworthy that intraperitoneal administration of the CD10 enzymatic inhibitors phosphoramidon and thiorphan significantly inhibited *in vivo* tumor growth of CD10-A375 cells (Fig 2E). These results suggest that CD10 functions to promote tumor cell proliferation, tumorigenicity, and tumor development by its enzymatic activity. In a cell migration assay, cell migration was significantly slower in CD10-A375 than in mock-A375. Similarly, cell invasion assay revealed that CD10-A375 showed decreased cell invasion. These results suggest that, upon acquiring CD10 expression, A375 melanoma cells undergo substantial changes in their biological behavior.

Regarding the effect of CD10 on tumor cell migration or invasion, conflicting results have been reported in different types of cancers. Ikenaga et al. reported that CD10+ pancreatic stellate cells promoted tumor cell invasion through MMP-3 secretion and thereby ECM degradation [13]. In bladder cancer, increased expression of CD10 in tumor and stromal cells of bladder carcinoma is strongly correlated with tumor progression, invasion and metastasis [7, 27]. In addition, Lee et al. [28] have recently published the association of CD10 over-expression in esophageal squamous cell carcinoma (ESCC) cells with activity of the transcriptional factor Twist 1, known inducers of epithelial mesenchymal transition (EMT) in ESCC [29]. On the other hand, multiple studies have shown the inhibitory effect of CD10 on tumor cell invasion; we previously reported that CD10 expressed by fibroblast inhibits invasive potency of squamous cell carcinoma cells by diminishing substance P levels in the tumor microenvironment [11]; in ovarian cancer, CD10 overexpression in ovarian carcinoma cells resulted in a significant decrease in cell proliferation and invasiveness with a reduction in the concentration of endothelin-1 in the conditioned medium [23]; in prostate cancer model, CD10 has been shown to block PI3K interaction with FAK by competitive binding, leading to decreased FAK phosphorylation and cell migration and adhesion [30, 31]. These findings suggest a negative regulation of cell migration by CD10. Thus, the molecular mechanisms underlying the role of CD10 in cancer invasion remain largely unclear, and it might be possible that CD10 functions differently on cell invasion in different types of cancers.

Multiple studies have suggested a ‘‘go or grow” dichotomy in cancer denoting a negative correlation between invasive and proliferative phenotypes in tumors [32–36], and that gene expression signatures can classify tumor classes and predict patient survival [36–38]. For example, cancers with the proliferation signature, characterized by p53 and PTEN inactivation and concomitant Myc activation, correlates with poor outcome in lung, prostate, breast and brain cancer, whereas remodeling signature, characterized by RAS, HIF-1α and NFκB activation, increases mortality rates in colorectal and ovarian cancer [39, 40]. Hoek et al. [41] presented a very interesting model for melanoma progression, describing that melanoma cells undergo transcriptional signature switching *in vivo* from a proliferative to an invasive state and *vice versa* during melanoma progression and metastasis. They used proliferative-signature melanoma cells and invasive-signature melanoma cells and observed that the former exhibited more rapid growth *in vitro* and *in vivo* than the latter. Studies on other cancers [42, 43] also demonstrated that, although they exhibited lower invasiveness, tumor cells that did not exhibit EMT showed more rapid local tumor growth and higher metastatic potential than those that did. CD10 might be the proliferative-signature gene in melanoma since it promoted cell proliferation *in vitro*, tumor growth *in vivo*, and resistance to apoptosis, but decreased cell motility.

In summary, we found here that CD10-transfected A375 human melanoma cells have greater cell proliferation ability, tumorigenicity, and resistance to anticancer drugs than mock-A375 cells and that CD10-A375 has a gene expression profile that promotes tumor progression. From our previous and current studies, we propose that CD10 be considered as a new target candidate for the treatment of melanoma.
